# Supplementary material for: Retrograde signals control dynamic changes to the chromatin state at photosynthesis-associated loci
Source: Nat Commun. 2025 Jul 15;16:6527. doi: 10.1038/s41467-025-61831-w (PMC12264055; doi:10.1038/s41467-025-61831-w)
Supplement: Supplementary file 1 — Supplementary Information [file 41467_2025_61831_MOESM1_ESM.pdf]

Supplemental Figure 1

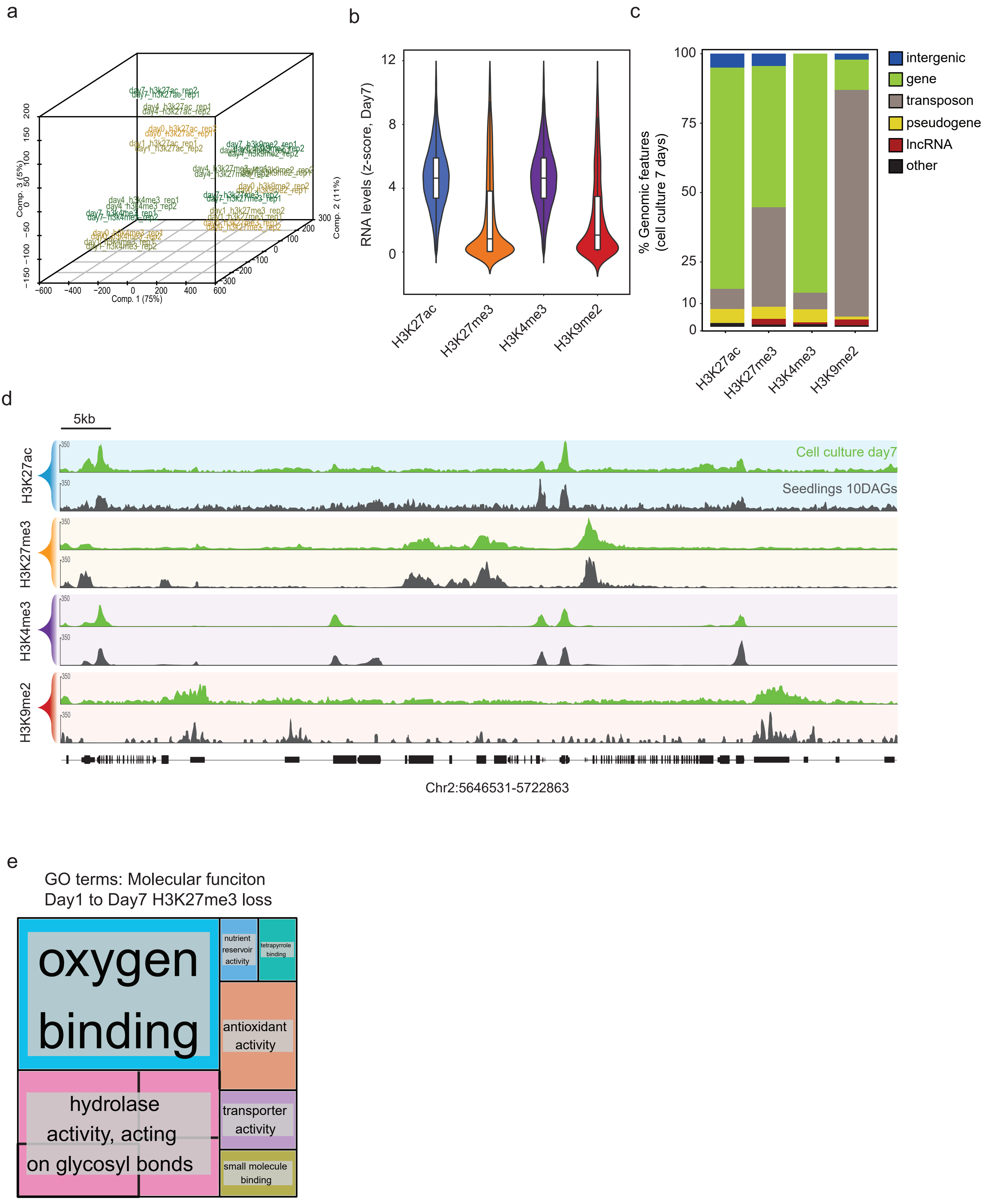

Supplementary Figure 1. ChIP-seq overview. a. Principal component analysis displaying each histone-PTM after H3 normalization in all individual replicates. **b.** Normalized RNA expression levels of genes annotated to histone PTM ChIP regions at *Arabidopsis* Day7 cell culture. Data retrieved from Dubreuil et. al 2018. **c.** Stacked bar plot showing the annotation of *Arabidopsis* Day7 cell culture peaks histone PTMs to different genomic features, according to Araport11. **d.** ChIP-seq visualization tracks of histone PTMs occupancy of *Arabidopsis* Day7 cell culture and published 10DAGs seedling data. A normalized signal is indicated on the y-axis. A scale bar is indicated. **e.** GO ontology terms (Molecular function) found enriched in the genes where H3K27me3 was found depleted using TreeMap adapted from REVIGO.

Supplemental Figure 2

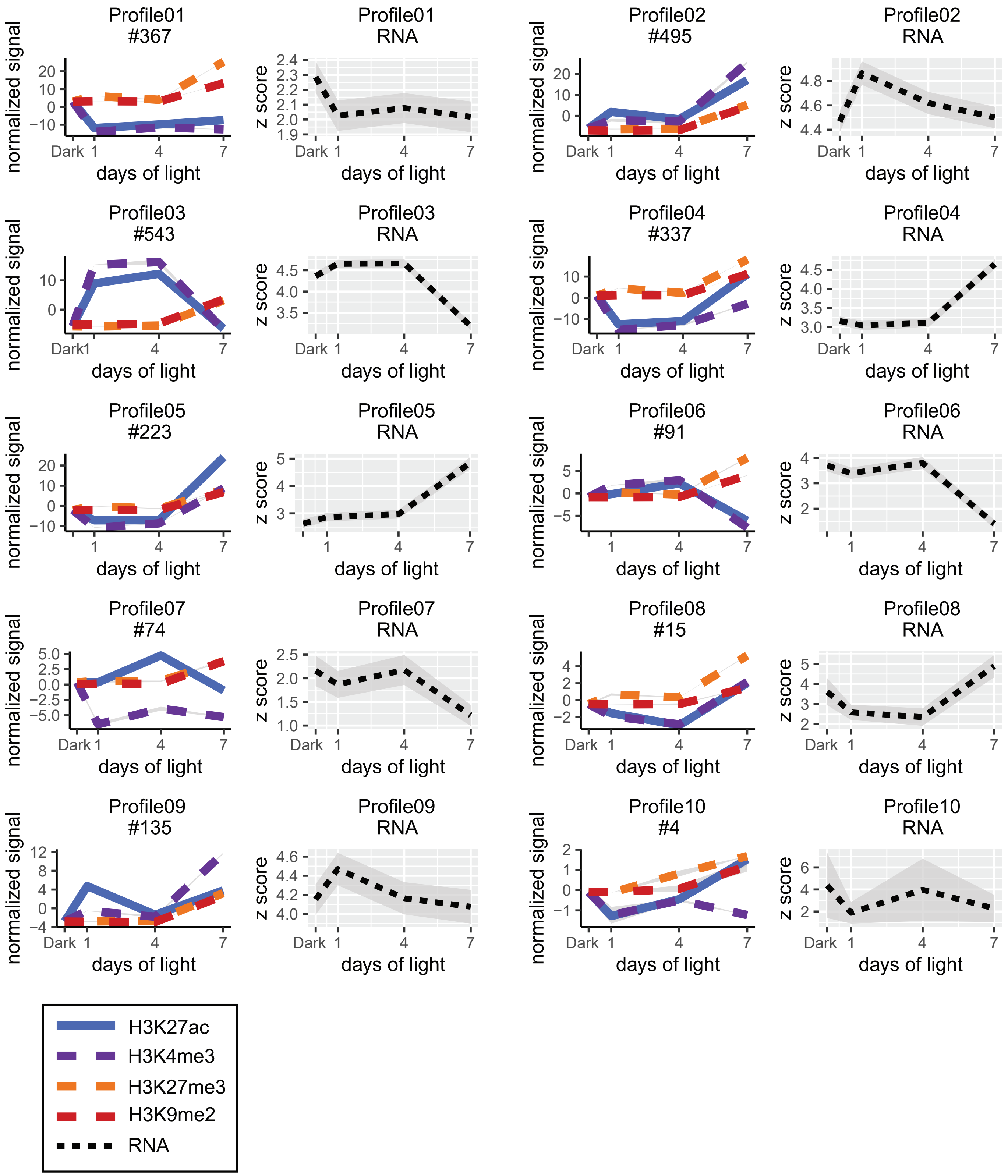

Supplementary Figure 2. Histone H3K27ac temporal trajectories. Paired graphs of normalized ChIP-seq signal and RNA expression at DERs following different H3K27ac enrichment profiles. Number of regions following each profile is reported (#). Profile5 was used in further analysis. RNA-seq data was retrieved from Dubreuil et. al 2018.

Supplemental Figure 3

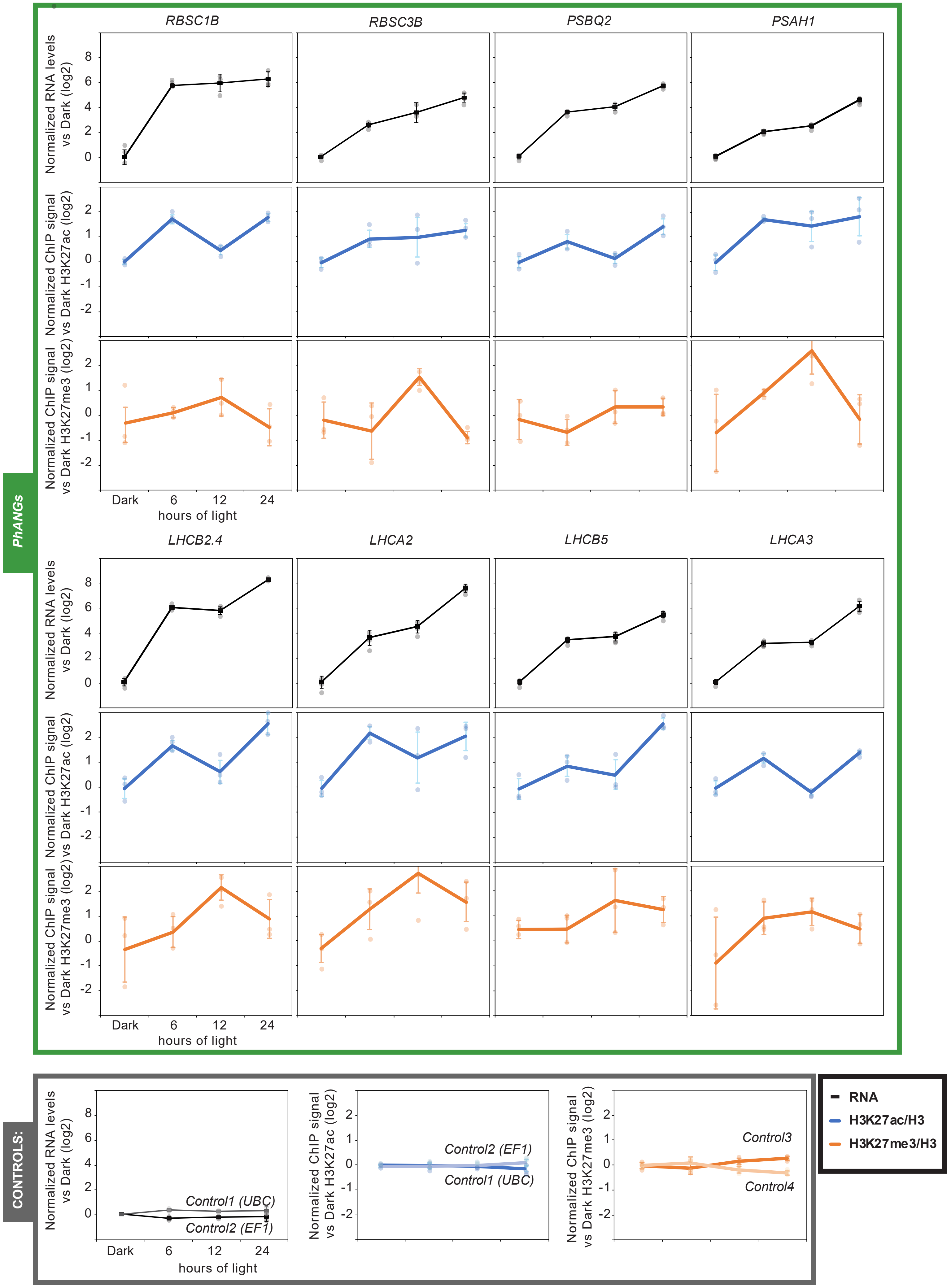

Supplementary Figure 3. Histone PTM evaluation in de-etiolation seedlings by ChIP-qPCR. Normalized ChIP-qPCR values for H3K27ac/H3 and H3K27me3/H3 ratios at eight *PhANG* loci plus four reference genes (Control1 *UBC*,Control2 *EF1* for H3K27ac plus Control3 and Control4 for H3K27me3) used in Figure 2g. ChIP was performed at 4 timepoints, Dark, 6, 12 or 24 hours following light exposure of dark-grown seedlings. Values were normalized by control region signal and to the Dark condition. Three independent biological replicates were used, and the individual data points are indicated by circles.

Supplemental Figure 4

a

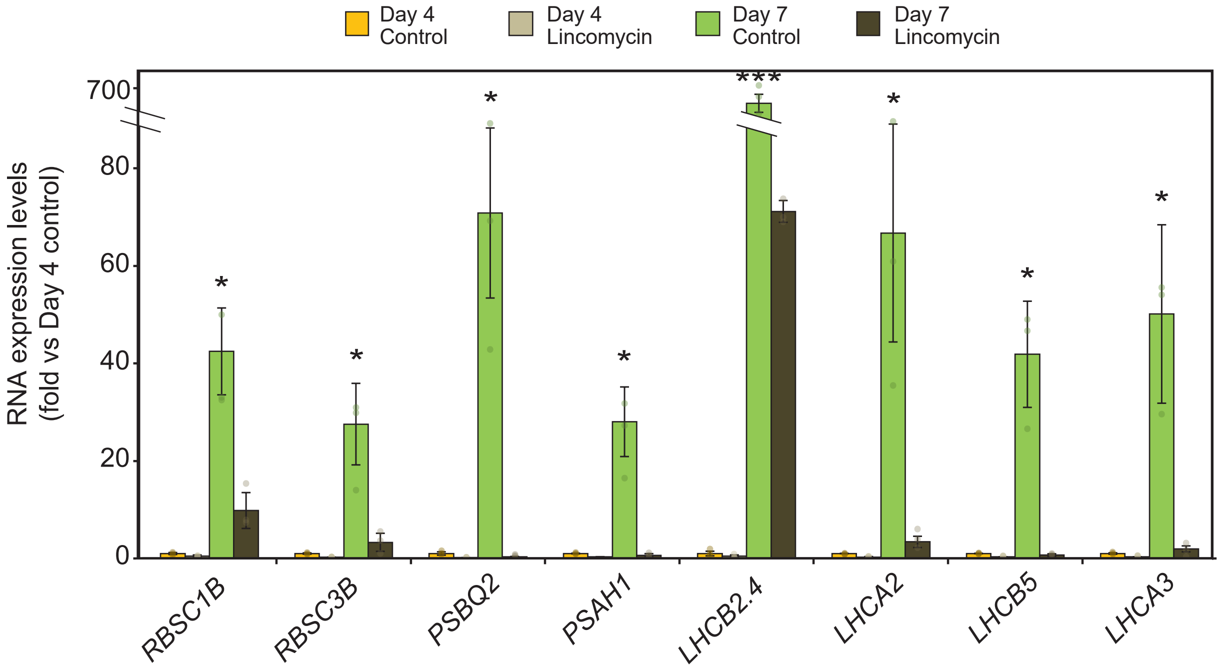

b

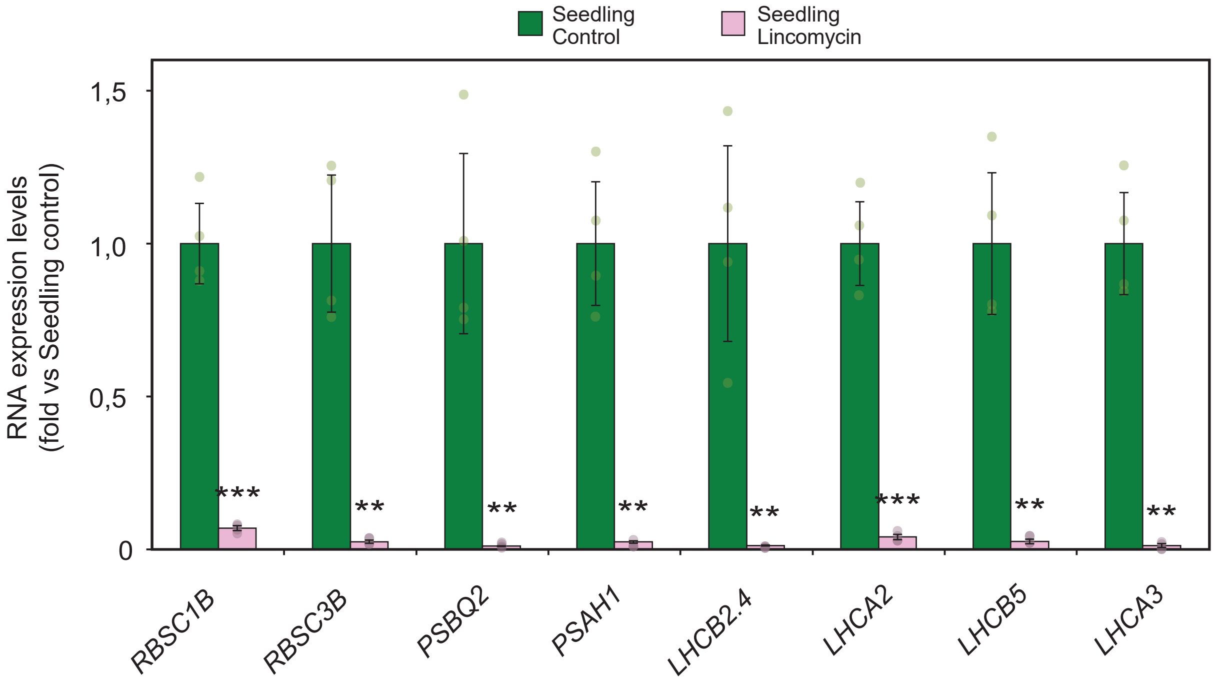

Supplementary Figure 4. Lincomycin repression *PhANG* expression in cell culture and seedlings. a. Expression levels of eight *PhANGs* in Day4 or Day7 *Arabidopsis* cell culture samples treated with/without lincomycin since Day1. Values were normalized to the Day4 Control condition. Two reference genes (*UBC*, *EF1*) were used for normalization. Three independent biological replicates were used, and significance was assessed by unpaired Student *t*-test (*, $p<0.05$). **b.** RNA expression levels of eight *PhANGs* in 7-day-old Col-0 *Arabidopsis* seedlings grown with/without lincomycin. Values normalized to seedlings grown without lincomycin. Two reference genes (*UBC*, *EF1*) were used for normalization. Three independent biological replicates were used, and significance was assessed by unpaired Student *t*-test, *, $p<0.05$. The individual data points are indicated by circles.

Supplemental Figure 5

a

Predicted TF	Motif	% of targets	P-value
**H3K27ac Day 4-7 increased DERs:**			
GATA20(C2C2gata)	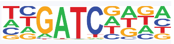	75,27	1,00E-03
ANAC038(NAC)	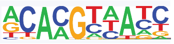	63,10	1,00E-03
AT5G61620(MYBrelated)	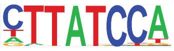	47,37	1,00E-04
SPL15(SBP)	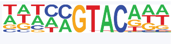	37,93	1,00E-04
HY5(bZIP)	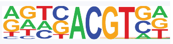	29,02	1,00E-08
RVE(MYBrelated)	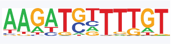	20,06	1,00E-16
AT1G76870(Trihelix)	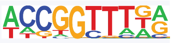	19,72	1,00E-13
REF6(C2H2 zfn)	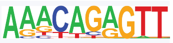	7,35	1,00E-13
**H3K4me3 Day 4-7 increased DERs:**			
GATA15(C2C2gata)	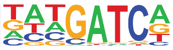	63,24	1,00E-06
GLK1/2(G2like)	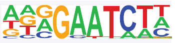	61,51	1,00E-03
ANAC057(NAC)	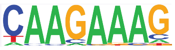	54,81	1,00E-16
ANAC045(NAC)	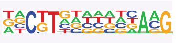	53,42	1,00E-05
AT5G05550(Trihelix)	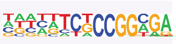	49,83	1,00E-03
REF6	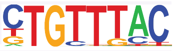	45,65	1,00E-03
LEC2(AP2/B3-like)	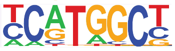	45,26	1,00E-16
AT1G71450(AP2EREBP)	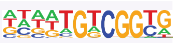	42,14	1,00E-03
AT1G69570(C2C2-Dof)	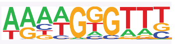	22,10	1,00E-16
**H3K27me3 Day 1-4 decreased DERs:**			
GATA20(C2C2gata)	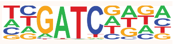	78,05	1,00E-04
ATAF1(NAC)	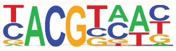	66,96	1,00E-08
At1g75490(AP2EREBP)	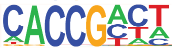	57,25	1,00E-04
ANAC045(NAC)	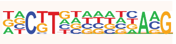	52,32	1,00E-04
RAX1(R2R3-MYB)	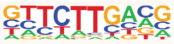	50,58	1,00E-025
VAL1(B3 domain)	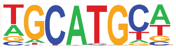	41,97	1,00E-11
G-box	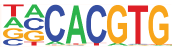	37,18	1,00E-07
bZIP50(bZIP)	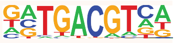	32,08	1,00E-04

b

Predicted TF	Motif	% of targets	P-value
**Overlap H3K27me3-H3K27ac**			
ANAC045(NAC)	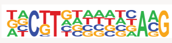	60,68	1,00E-05
FUS3(ABI3VP1)	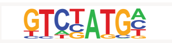	50,46	1,00E-09
EPR1(MYBrelated)	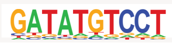	34,06	1,00E-08
MYB3R1(MYB)	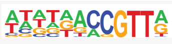	29,72	1,00E-03
At4g31060(AP2EREBP)	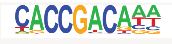	21,36	1,00E-04
GLK1/2(G2like)	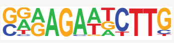	11,15	1,00E-11

c

**Profile5 H3K27ac**			
AT5G60130(ABI3VP1)	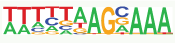	74,89	1,00E-02
ANAC038(NAC)	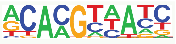	68,16	1,00E-02
AT5G61620(MYBrelated)	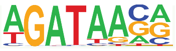	62,33	1,00E-02
ZAT6(homeobox)	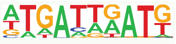	60,54	1,00E-02
ATHB23(ZFHD)	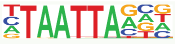	48,43	1,00E-02
ANAC016(NAC)	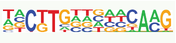	30,94	1,00E-02
G-box	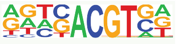	29,60	1,00E-02

Begränsad delning

Supplementary Figure 5. De novo motifs found at DERs. a. Enriched sequence motifs identified by the HOMER differential motif discovery algorithm for H3K27ac and H3K4me3 DERs at Day4 to Day7 transition and H3K27me3 DERs at Day1 to Day4 transition. **b.** Enriched sequence motifs identified by the HOMER differential motif discovery algorithm for H3K27me3 decreased DERs from Day1 to Day4, that overlap with H3K27ac increased DERs from Day4 to Day7. **c.** Enriched sequence motifs identified by the HOMER differential motif discovery algorithm for DERs following H3K27ac Profile5, from Fig 2A. Combined known and *de novo* HOMER outputs are reported.

Supplemental Figure 6

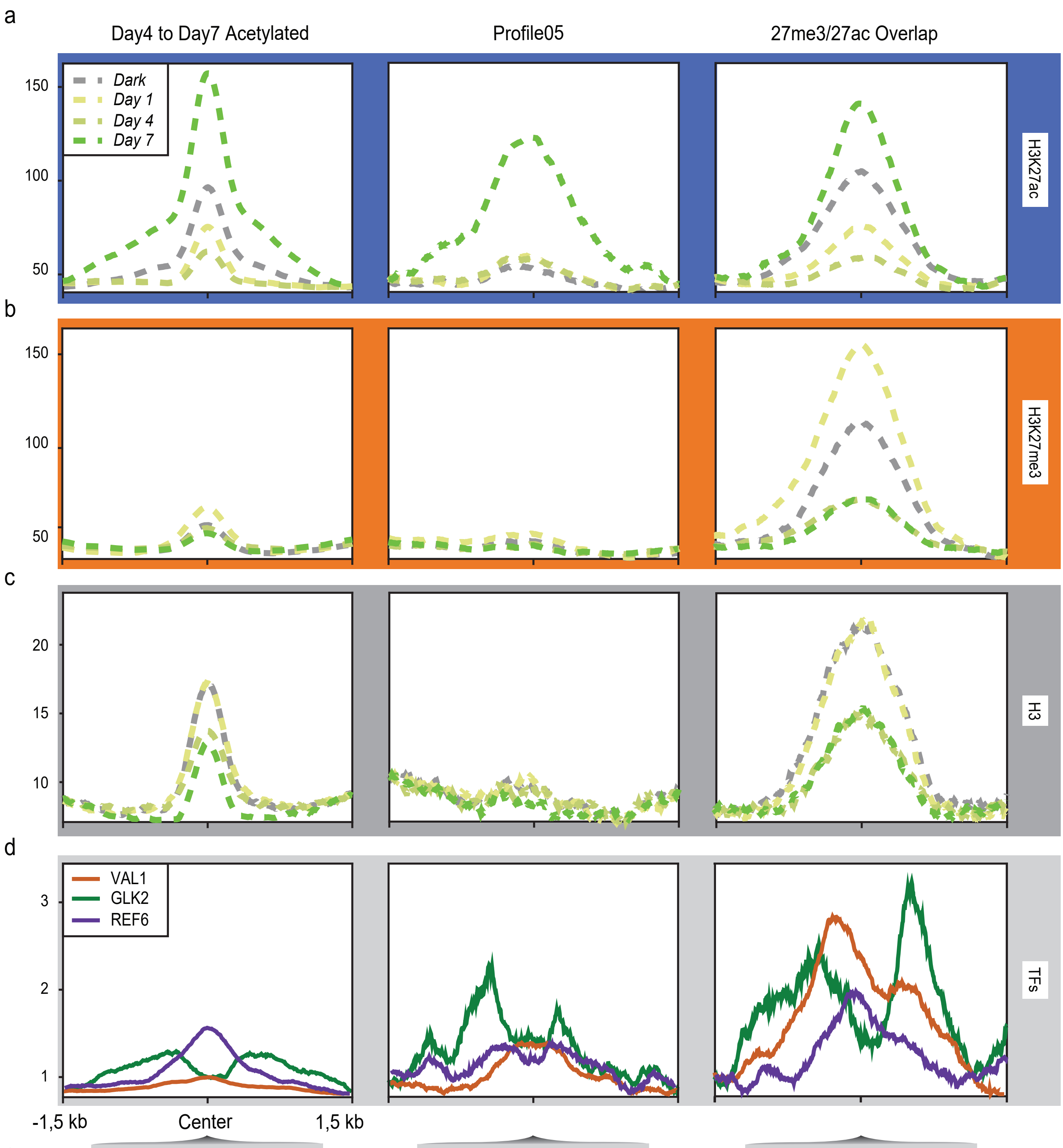

e

AT5G38430	RBCS1B
AT5G38410	RBCS3B
AT1G79040	PSBR
AT3G27690	LHCB2.4
AT2G30570	PSBW
AT4G02770	PSAD-1
At2g05070	LHCB2.2
AT3G59400	GUN4
AT4G12800	PSAL
At4g05180	PSBQ2
AT2G05100	LHCB2.1
AT3G61470	LHCA2
AT4G10340	LHCB5
AT1G61520	LHCA3
AT3G16140	PSAH-1
AT4G27440	PORB

AT5G38410	RBCS3B
AT5G38430	RBCS1B
AT1G79040	PSBR
AT3G27690	LHCB2.4
AT2G30570	PSBW
AT4G02770	PSAD-1

AT5G38410	RBCS3B
AT5G38430	RBCS1B
AT1G79040	PSBR
AT3G27690	LHCB2.4
AT2G05070	LHCB2.2
AT3G59400	GUN4
AT4G12800	PSAL
AT4G05180	PSBQ2
AT2G05100	LHCB2.1
AT3G61470	LHCA2
AT4G10340	LHCB5

Begränsad delning

Supplementary Figure 6. Genome-wide characterization of loci of interest. a-c. Metagene profile of histone PTM ChIP-seq data from this study at different gene subsets. Signal is centred to each DER region middle point and plotted within a ±1.5 kb of the window. Line colours indicate the ChIP-seq time-points. **d.** Metagene profile of transcription factors GLK2, VAL1 and REF6 ChIP-seq published data (Supplementary Data 1) at different gene subsets. The same scale is maintained in the 3 subsets at each row. **e.** *PhANGs* annotated to each subset. Colours are used to indicate overlaps.

Supplemental Figure 7

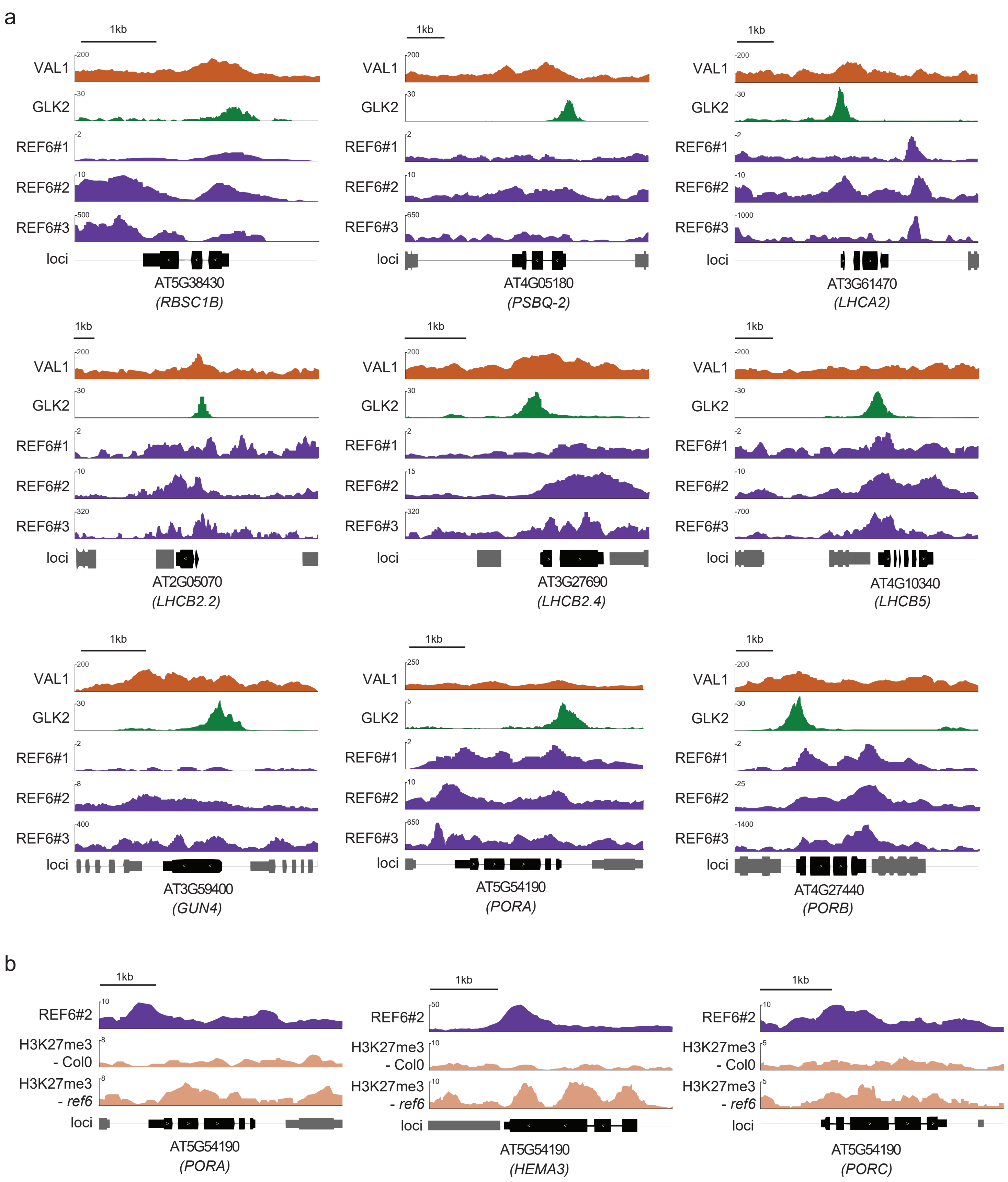

Supplementary Figure 7. Visual examples of loci of interest. a. ChIP-seq visualization tracks of VAL1, GLK2 and REF6 (from 3 independent studies) occupancy from published seedling data (Supplementary Data 1) at several *PhANG* loci evaluated in this study. The normalized signal is indicated on the y-axis. A 1 kb scale bar is indicated in each locus. **b.** ChIP-seq visualization tracks of REF6 and H3K27me3 in Col0 and ref6 mutant line (from the same study [45]).

Supplemental Figure 8

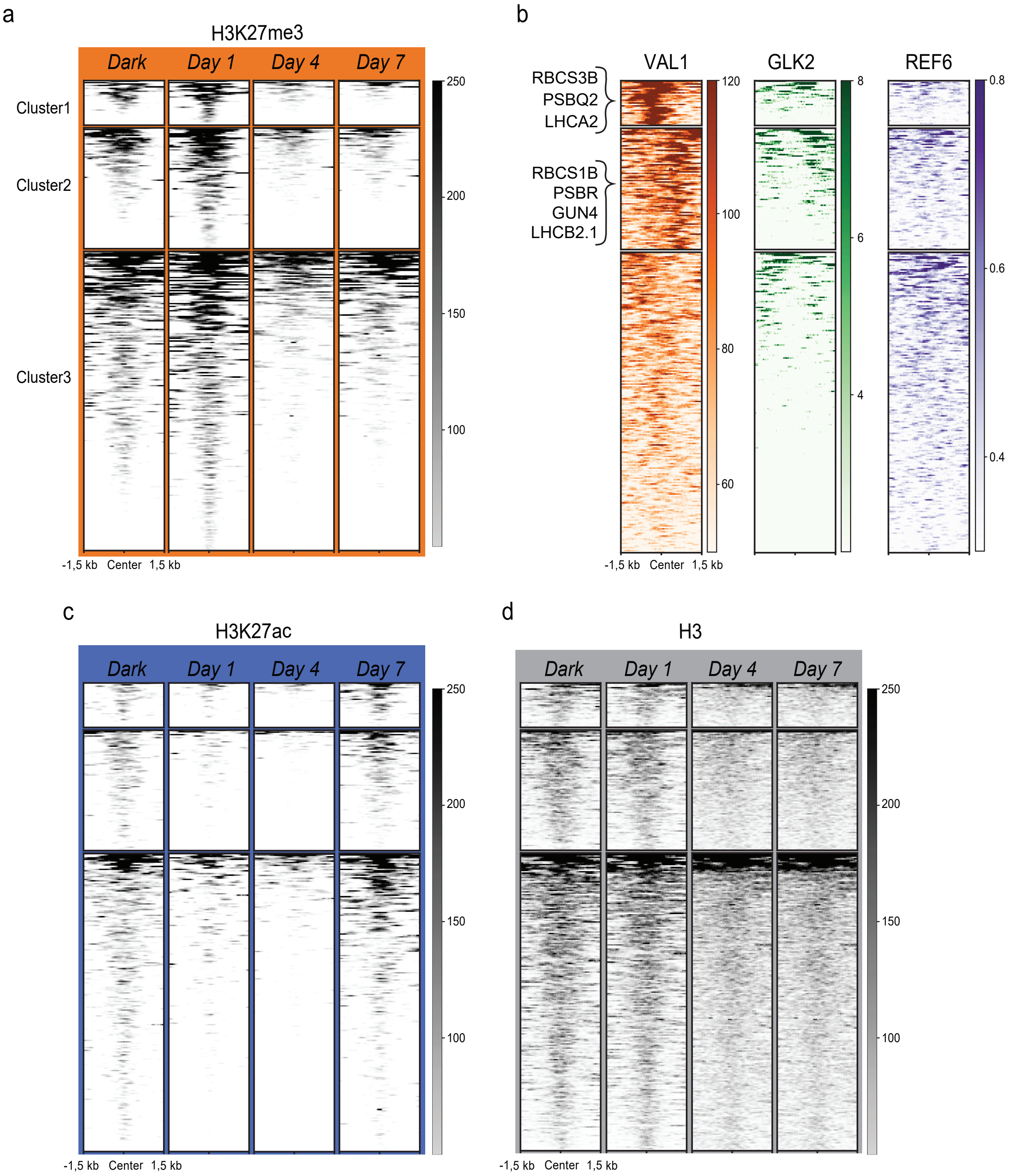

Begränsad delning

Supplementary Figure 8. H3K27me3/H3K27ac subset genomic characterization. ChIP-seq heatmaps at the ”overlap” subset (Fig. 2d). Peaks are ranked by VAL1 ChIP signal. 3 clusters were identified to be distinct in VAL1 occupancy and heatmaps were split and ranked independently by VAL1 signal. Each row in a cluster represents one peak with a ±1.5 kb window centred for each DER region middle point. Scales are displayed in the y-axis. **a.** H3K27me3 ChIP-seq data from this study. **b.** Published ChIP-seq data for VAL1, GLK2 and REF6. *PhANGs* annotated to regions at cluster1 and cluster2 are displayed. **c.** H3K27ac ChIP-seq data from this study. **d.** H3 ChIP-seq data from this study.

Supplemental Figure 9

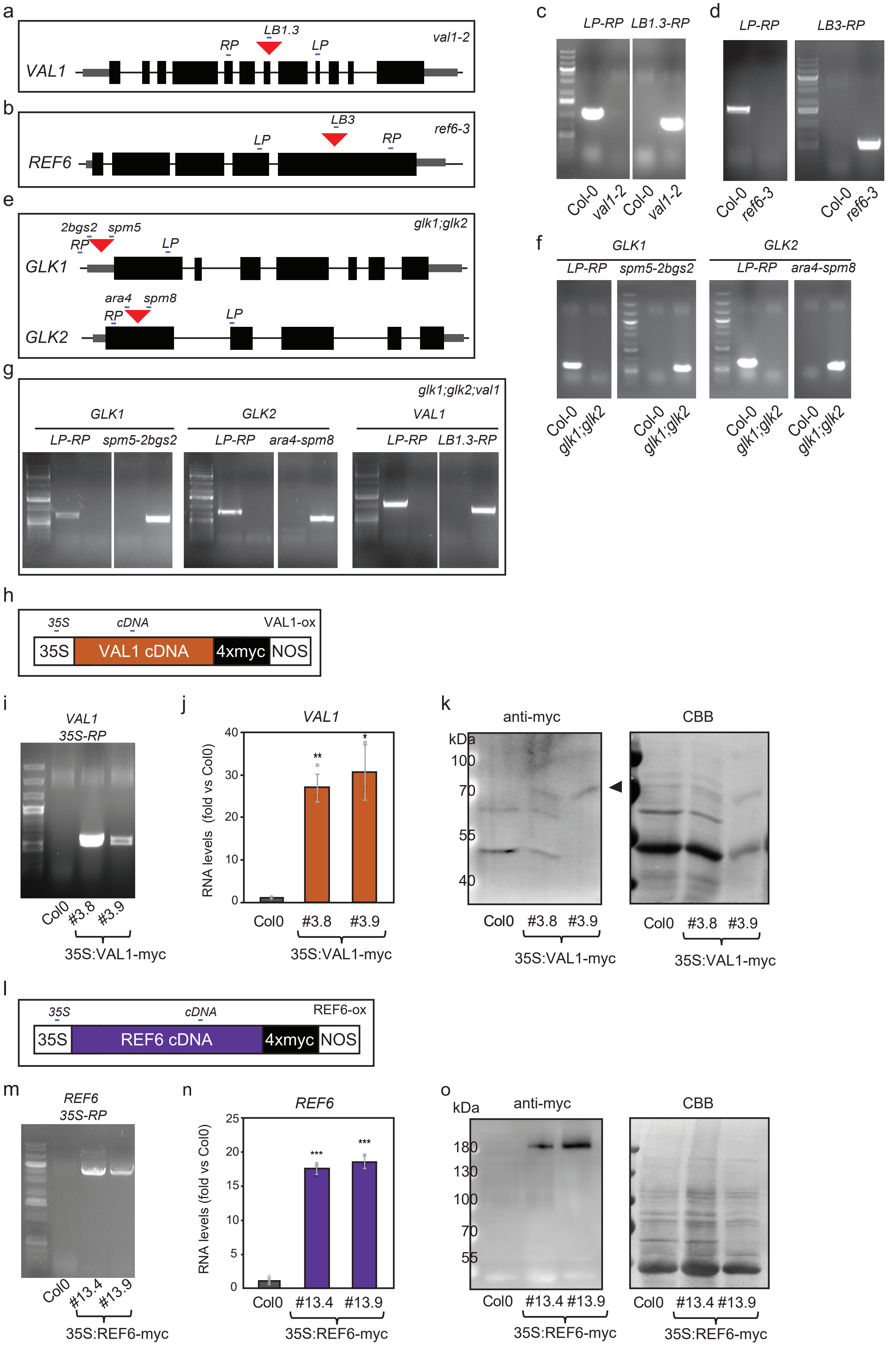

Supplementary Figure 9. Generation and verification of mutant and overexpressing lines. **a,b,e.** Schematic representation of T-DNA insertion mutants and overexpressing lines used in this study. Red triangles depict T-DNA insertions. LP and RP denotes genotyping primer positions. **c.** PCR genotyping of *val1-2* T-DNA line. **d.** PCR genotyping of *ref6-3* T-DNA line. **f.** PCR genotyping of *glk1;glk2* T-DNA line. g. PCR genotyping of the *glk1;glk2;val1* triple mutant. **h,l.** Schematic representation of overexpression constructs generated in this study. **i.** PCR genotyping of VAL1-ox lines. **j.** Expression levels of *VAL1* transcripts in Col-0 and VAL1-ox lines. Values normalized to Col-0 control line. Two reference genes (UBC, EF1) were used for normalization. Three independent biological replicates were used and significance was assessed in triplicates by unpaired Student t-test (***, $p<0.001$). The individual data points are indicated by circles. **k.** Western Blot detection of protein in VAL1-ox lines using anti-Myc antibodies. Coomassie blue staining (CBB) as loading control. **m.** PCR genotyping of REF6-ox lines**. n.** Expression levels of *REF6* transcripts in Col-0 and REF6-ox lines. Values normalized to Col-0 control line. Two reference genes (UBC, EF1) were used for normalization. Three independent biological replicates were used and significance was assessed in triplicates by unpaired Student t-test (***, $p<0.001$). The individual data points are indicated by circles. **o.** Western Blot detection of protein in REF6-ox lines using anti-Myc antibodies. Coomassie blue staining (CBB) as loading control.

Supplemental Figure 10

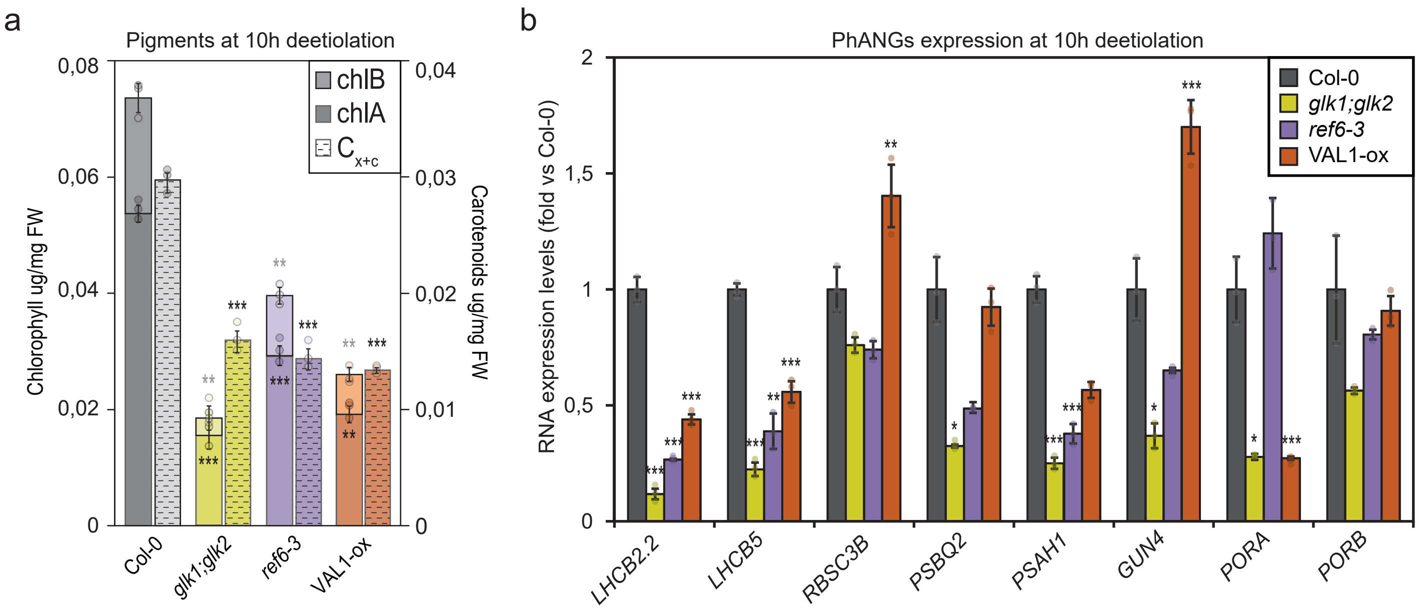

Supplementary Figure 10. Phenotypic characterization of *glk1;glk2, ref6* and VAL1ox at 10h de-etiolation. a. Pigment content of 3-day dark-grown seedlings exposed to 10 hours of light. Chlorophylls and carotenoid concentration quantified in µg/g FW. Three independent biological replicates were used and significance was assessed by unpaired Student t-test (**p<0.01;***p<0.001). The individual data points are indicated by circles **b.** Expression levels of eight *PhANGs* in Col-0, *glk1;glk2* and *VAL1-ox* following 10 hours of exposure to light. Values normalized to Col-0. Two reference genes (*UBC, EF1*) were used for normalization. Three independent biological replicates were used and significance was assessed in triplicates by unpaired Student *t*-test (*, p<0.05; **,p<0.005, ***, p<0.001). The individual data points are indicated by circles.

Supplemental Figure 11

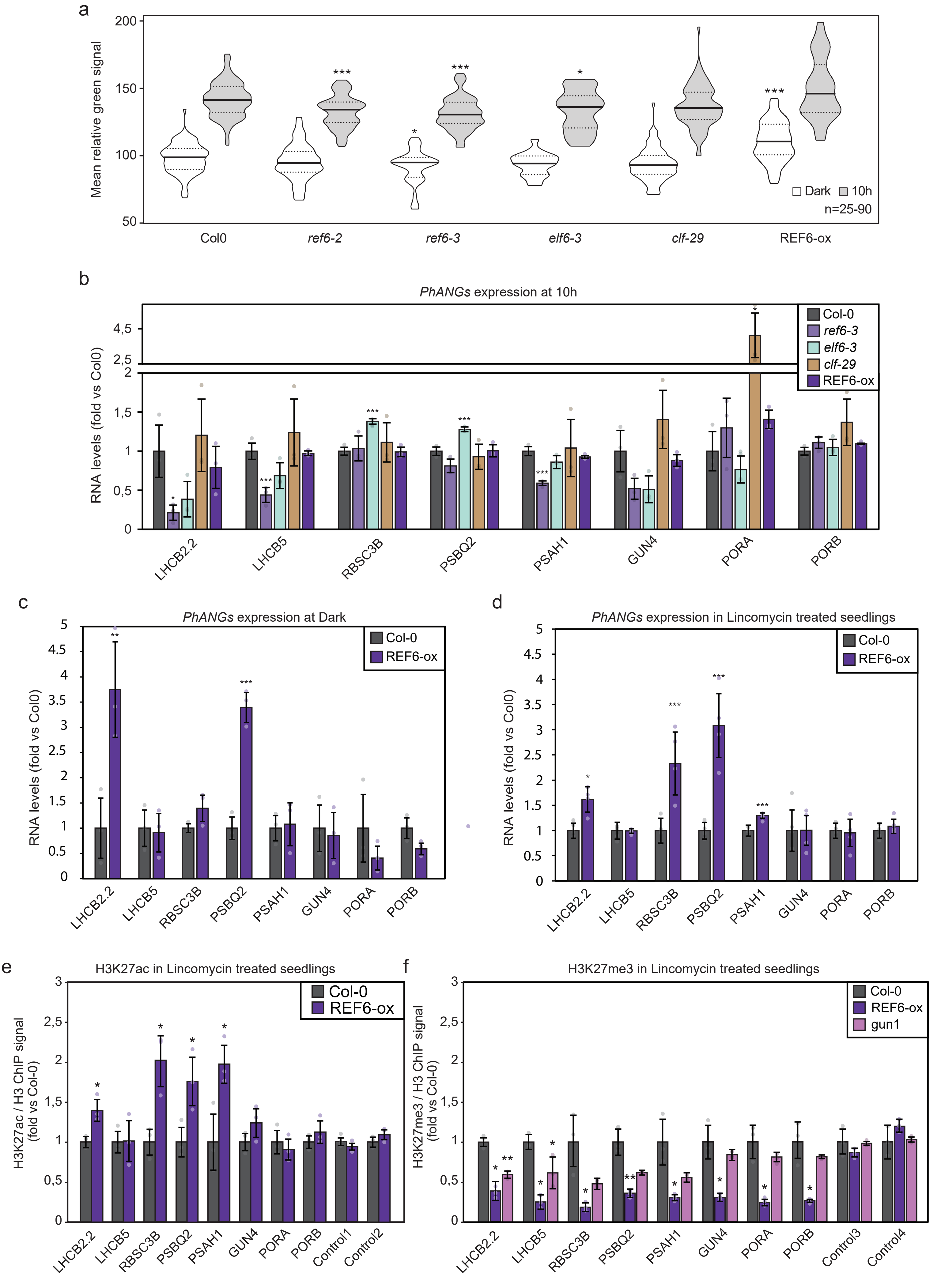

Supplementary Figure 11. REF6 is involved in seedling de-etiolation by its role in chromatin regulation. a. Quantification of green levels after image processing of 3-day-old dark grown seedlings, at dark and following 10 hours exposure to light. RGB channels were split and the green value level was measured in cotyledons. Significance was assessed in n>25 by the One-way ANOVA test followed by Dunnett post-hoc test for comparison (*, p<0,05; ***, p<0,001) **b.** Expression levels of eight *PhANGs* in Col-0 and mutant and transgenic lines of 3-day-old dark-grown seedlings following 10h light exposure. Values normalized to Col-0. Two reference genes (*UBC, EF1*) were used for normalization. Three independent biological replicates were used and significance was assessed in triplicates by unpaired Student t-test (*, p<0,05; ***, p<0,001). **c.** Expression levels of eight *PhANGs* in Col-0 and *REF6* overexpression lines in 3-day-old dark-grown seedlings at dark. Values were normalized to Col-0 and two reference genes (*UBC, EF1*) were used for normalization. Three independent biological replicates were used and significance was assessed by unpaired Student *t*-test (**, p<0.005; ***, p<0.001). **d.** Expression levels of eight *PhANGs* in Col-0 and *REF6* overexpression lines grown on lincomycin and constant light for 7 days. Values were normalized to Col-0 and two reference genes (*UBC, EF1*) were used for normalization. Three independent biological replicates were used, and significance was assessed by unpaired Student *t*-test (*, p<0.05; ***, p<0.001). **e.** ChIP-qPCR values for H3K27ac/H3 ratio at eight *PhANG* loci plus two control regions. Col-0 and REF6-ox were grown on lincomycin in the light for 7 days. Values were normalized by control region signal and to Col-0 grown on lincomycin. Three independent biological replicates were used and the significance was assessed between triplicates by unpaired Student *t*-test (*p<0.05). **f.** ChIP-QPCR values for H3K27me3/H3 ratio at eight *PhANG* loci plus two control regions. Col-0, REF6-ox and *gun1* seedlings were grown on lincomycin in the light for 7 days. Values were normalized by control region signal and to Col-0 grown on lincomycin. Three independent biological replicates were used, and the significance was assessed between triplicates by unpaired Student *t*-test (*p<0.05; **, p<0.005). In the bar diagrams the individual data points are indicated by circles.

Supplemental Figure 12

a

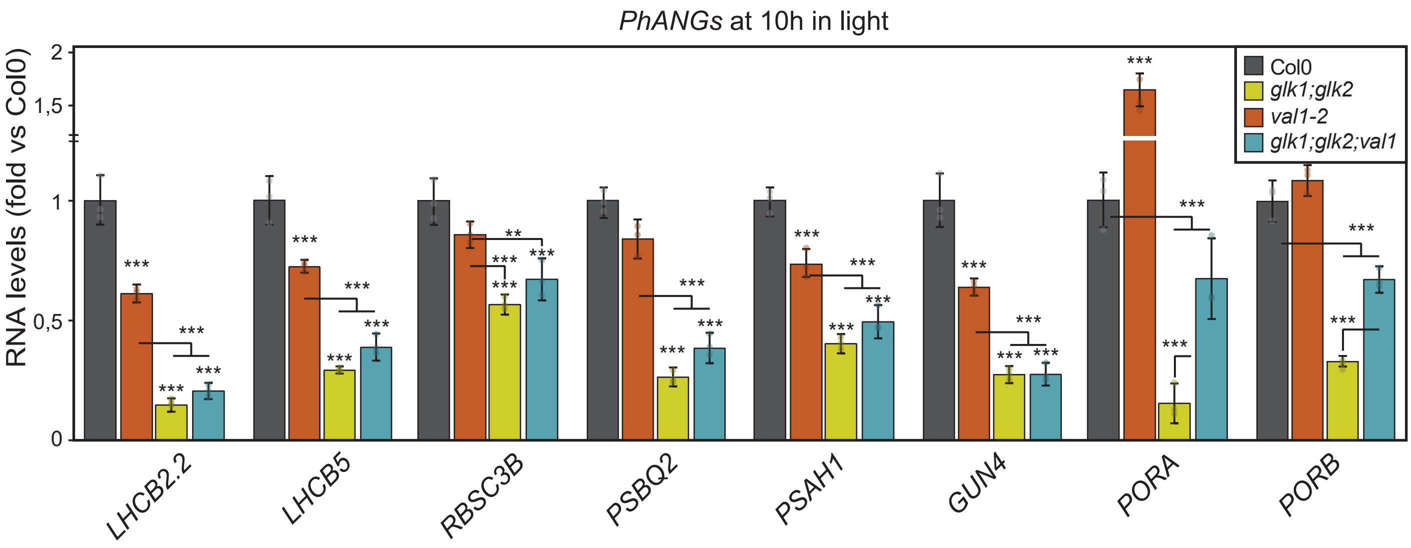

b

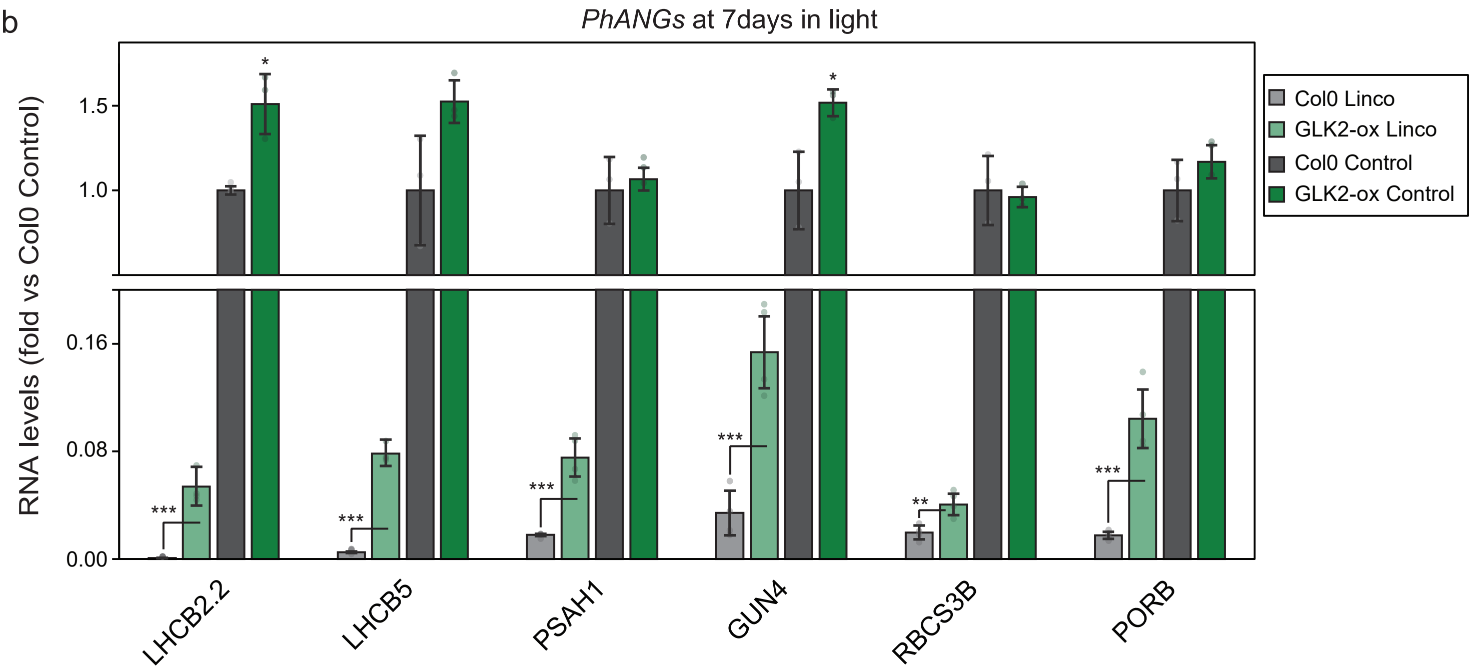

Supplementary Figure 12. VAL1 and GLK2 regulate *PhANG* expression. a. Expression levels of eight *PhANGs* in Col-0 and mutant and transgenic lines of 3-day-old dark-grown seedlings after 10h light exposure. Values normalized to Col-0. Two reference genes (UBC, EF1) were used for normalization. Three independent biological replicates were used, and the significance was assessed in triplicates by unpaired Student t-test (**,p<0,005, ***, p<0,001). **b.** Expression levels of six *PhANGs* in Col-0 and mutant and transgenic lines of 7-day-old seedlings grown in constant light and with or without lincomycin. Values normalized to Col-0 Control (without lincomycin). Two reference genes (*UBC, EF1*) were used for normalization. Three independent biological replicates were used, and the significance was assessed in triplicates by unpaired Student t-test (*, p<0.05; ***, p<0.001). In a and b, the individual data points are indicated by circles.
