# Supplementary material for: Retrograde signals control dynamic changes to the chromatin state at photosynthesis-associated loci
Source: Nat Commun. 2025 Jul 15;16:6527. doi: 10.1038/s41467-025-61831-w (PMC12264055; doi:10.1038/s41467-025-61831-w)
Supplement: Supplementary file 7 — Reporting Summary [file 41467_2025_61831_MOESM7_ESM.pdf]

nature portfolio

Corresponding author(s): Åsa Strand, Marti Quevedo

Last updated by author(s): 17/6/25

Reporting Summary

Nature Portfolio wishes to improve the reproducibility of the work that we publish. This form provides structure for consistency and transparency in reporting. For further information on Nature Portfolio policies, see our Editorial Policies and the Editorial Policy Checklist.

Statistics

For all statistical analyses, confirm that the following items are present in the figure legend, table legend, main text, or Methods section.

n/a	Confirmed	
☐	☒	The exact sample size (*n*) for each experimental group/condition, given as a discrete number and unit of measurement
☐	☒	A statement on whether measurements were taken from distinct samples or whether the same sample was measured repeatedly
☐	☒	The statistical test(s) used AND whether they are one- or two-sided<br>*Only common tests should be described solely by name; describe more complex techniques in the Methods section.*
☒	☐	A description of all covariates tested
☒	☐	A description of any assumptions or corrections, such as tests of normality and adjustment for multiple comparisons
☐	☒	A full description of the statistical parameters including central tendency (e.g. means) or other basic estimates (e.g. regression coefficient) AND variation (e.g. standard deviation) or associated estimates of uncertainty (e.g. confidence intervals)
☐	☒	For null hypothesis testing, the test statistic (e.g. *F*, *t*, *r*) with confidence intervals, effect sizes, degrees of freedom and *P* value noted<br>*Give P values as exact values whenever suitable.*
☐	☒	For Bayesian analysis, information on the choice of priors and Markov chain Monte Carlo settings
☒	☐	For hierarchical and complex designs, identification of the appropriate level for tests and full reporting of outcomes
☒	☐	Estimates of effect sizes (e.g. Cohen's *d*, Pearson's *r*), indicating how they were calculated

Our web collection on statistics for biologists contains articles on many of the points above.

Software and code

Policy information about availability of computer code

Data collection	qPCR data collection was performed either in Biorad CFX Maestro Software or Roche LightCycler 480 Software (version 1.5..1.52. SP3)
Data analysis	FastQC (0.10.1)<br>bwa/0.7.17<br>picard/2.22.3<br>trimmomatic/0.36<br>MACS3/3.0.0b3<br>samtools/1.10<br>bedtools/2.29.2<br>R/4.2.0<br>STAR/2.7.9a<br>HOMER/4.11.1<br>GOFER /0.11<br>deepTools/3.5.6<br>python (version 3.11.4)

For manuscripts utilizing custom algorithms or software that are central to the research but not yet described in published literature, software must be made available to editors and reviewers. We strongly encourage code deposition in a community repository (e.g. GitHub). See the Nature Portfolio guidelines for submitting code & software for further information.

Data

Policy information about availability of data

All manuscripts must include a data availability statement. This statement should provide the following information, where applicable:

- Accession codes, unique identifiers, or web links for publicly available datasets
- A description of any restrictions on data availability
- For clinical datasets or third party data, please ensure that the statement adheres to our policy

A detailed compilation of scripts and processing files can be found at: https://github.com/martiquevedo/arabidopsis-epi-greening

Research involving human participants, their data, or biological material

Policy information about studies with human participants or human data. See also policy information about sex, gender (identity/presentation), and sexual orientation and race, ethnicity and racism.

Reporting on sex and gender	n/a
Reporting on race, ethnicity, or other socially relevant groupings	n/a
Population characteristics	n/a
Recruitment	n/a
Ethics oversight	n/a

Note that full information on the approval of the study protocol must also be provided in the manuscript.

Field-specific reporting

Please select the one below that is the best fit for your research. If you are not sure, read the appropriate sections before making your selection.

☒ Life sciences ☐ Behavioural & social sciences ☐ Ecological, evolutionary & environmental sciences

For a reference copy of the document with all sections, see nature.com/documents/nr-reporting-summary-flat.pdf

Life sciences study design

All studies must disclose on these points even when the disclosure is negative.

Sample size	We have duplicate samples for the large ChIPseq experiment. For all other experiments we used three replicates except in Figure 4g and Figure 5c where duplicates were used as indicated in the legends.
Data exclusions	No data has been excluded.
Replication	The experiments were repeated once or twice as indicated in the legends.
Randomization	The biological replicates used in the study were all independent and selected randomly from a population.
Blinding	n/a

Reporting for specific materials, systems and methods

We require information from authors about some types of materials, experimental systems and methods used in many studies. Here, indicate whether each material, system or method listed is relevant to your study. If you are not sure if a list item applies to your research, read the appropriate section before selecting a response.

Materials & experimental systems

n/a	Involved in the study
☐	☒ Antibodies
☐	☒ Eukaryotic cell lines
☒	☐ Palaeontology and archaeology
☒	☐ Animals and other organisms
☒	☐ Clinical data
☒	☐ Dual use research of concern
☐	☒ Plants

Methods

n/a	Involved in the study
☐	☒ ChIP-seq
☒	☐ Flow cytometry
☒	☐ MRI-based neuroimaging

Antibodies

Antibodies used	Anti-H3 (Agrisera, Cat. AS10710), anti-H3K4me3 (Millipore, Cat. 17-614), anti-H3K27me3 (Active Motif, Cat. 39155), anti-H3K27ac (Abcam, Cat. ab4729) or anti-H3K9me2 (Diagenode, Cat. C15410060) antibodies.
Validation	The antibodies used were commercial antibodies and validation was performed by the company.

Eukaryotic cell lines

Policy information about cell lines and Sex and Gender in Research

Cell line source(s)	We used an Arabidopsis thaliana cell culture
Authentication	Dubreuil, C. et al. Establishment of Photosynthesis through Chloroplast Development Is Controlled by Two Distinct Regulatory Phases. Plant Physiol 176, 1199-1214 (2018).
Mycoplasma contamination	We have not tested the cell line for Mycoplasma contamibation
Commonly misidentified lines (See ICLAC register)	n/a

Dual use research of concern

Policy information about dual use research of concern

Hazards

Could the accidental, deliberate or reckless misuse of agents or technologies generated in the work, or the application of information presented in the manuscript, pose a threat to:

No	Yes
☒	☐ Public health
☒	☐ National security
☒	☐ Crops and/or livestock
☒	☐ Ecosystems
☒	☐ Any other significant area

Experiments of concern

Does the work involve any of these experiments of concern:

No	Yes
☒	☐ Demonstrate how to render a vaccine ineffective
☒	☐ Confer resistance to therapeutically useful antibiotics or antiviral agents
☒	☐ Enhance the virulence of a pathogen or render a nonpathogen virulent
☒	☐ Increase transmissibility of a pathogen
☒	☐ Alter the host range of a pathogen
☒	☐ Enable evasion of diagnostic/detection modalities
☒	☐ Enable the weaponization of a biological agent or toxin
☒	☐ Any other potentially harmful combination of experiments and agents

Plants

Seed stocks	Seeds were obtained from the European Nottingham Arabidopsis Stock Centre (NASC)
Novel plant genotypes	35S::VAL1:4xMYC (VAL1ox) and 35S::REF6:4xMYC (REF6ox) was developed first by cloning the coding sequences from VAL1 or REF6 into pGWB517 60 via Gateway cloning, using pENTR™/D-TOPO™ and pDONR221 vectors, respectively (Thermo Fisher Scientific). Secondly, plant transformation was performed by floral dipping protocol using Agrobacterium tumefaciens (GV3101)-mediated transfer 61. 35S:VAL1:4xMYC or 35S::REF6:4xMYC was transformed into Col-0. Primers used for genotyping and cloning can be found in Supplementary Table5.
Authentication	Supplementary Figure 6 shows the data from the genotyping and analysis of the plant lines used in this study.

ChIP-seq

Data deposition

☒ Confirm that both raw and final processed data have been deposited in a public database such as GEO.

☒ Confirm that you have deposited or provided access to graph files (e.g. BED files) for the called peaks.

Data access links<br>*May remain private before publication.*	A detailed compilation of scripts and processing files can be found at: https://github.com/martiquevedo/Quevedo_et_al_2025 or https://doi.org/10.5281/zenodo.15674768
Files in database submission	*Provide a list of all files available in the database submission.*
Genome browser session<br>(e.g. UCSC)	*Provide a link to an anonymized genome browser session for "Initial submission" and "Revised version" documents only, to enable peer review. Write "no longer applicable" for "Final submission" documents.*

Methodology

Replicates	*Describe the experimental replicates, specifying number, type and replicate agreement.*
Sequencing depth	*Describe the sequencing depth for each experiment, providing the total number of reads, uniquely mapped reads, length of reads and whether they were paired- or single-end.*
Antibodies	Anti-H3 (Agrisera, Cat. AS10710), anti-H3K4me3 (Millipore, Cat. 17-614), anti-H3K27me3 (Active Motif, Cat. 39155), anti-H3K27ac (Abcam, Cat. ab4729) or anti-H3K9me2 (Diagenode, Cat. C15410060) antibodies.
Peak calling parameters	*Specify the command line program and parameters used for read mapping and peak calling, including the ChIP, control and index files used.*
Data quality	*Describe the methods used to ensure data quality in full detail, including how many peaks are at FDR 5% and above 5-fold enrichment.*
Software	*Describe the software used to collect and analyze the ChIP-seq data. For custom code that has been deposited into a community repository, provide accession details.*
